# Relative Yield Loss Calculations in Wheat (*Triticum durum* Desf. cv. Camacho) due to Ozone Exposure

**DOI:** 10.1100/tsw.2010.26

**Published:** 2010-01-21

**Authors:** Daniel de la Torre

**Affiliations:** Universidad Politécnica de Madrid, Plant Biology Department, ETSI Agronomos, Madrid, Spain

**Keywords:** ozone exposure, ozone uptake, relative yield losses, wheat

## Abstract

In this work, an estimation of the relative yield losses of wheat due to ozone exposure is made by means of two approaches proposed by the CLRTAP (Convention on Long Range Transboundary Air Pollution): the exposure-response approach, which deals with the exposure of plants to ozone during a certain time, and the accumulated uptake approach, which, besides ozone exposure, deals with the velocity of absorption of the contaminant and the environmental factors that modulate that absorption. Once the relative yield losses are calculated by means of the two approaches, the aim is to establish which index (the exposure-response index or the accumulated uptake index) best characterizes the response of wheat plants to ozone. The relative yield losses are compared considering two watering regimes: well watered and nonwatered. The results obtained show that the relative yield losses in wheat due to ozone exposure are much more strongly linked to the real quantity of ozone absorbed by plants than to the environmental ozone exposure, which means that the accumulated uptake approach is much more realistic than the exposure-response approach. Relative yield loss estimations were higher in a crop with no watering; 3% of relative yield losses more than a crop watered until field capacity.

## INTRODUCTION

Tropospheric ozone has been shown to induce important yield losses in cereal crops[14,16,42]. A negative aspect of the exposure of cereal crops to ozone is yield loss. In wheat, this can be seen as decreasing spike numbers per surface unit, decreasing grain numbers per spike, and a decrease in grain weight, with the consequent decrease in spike weight and size[8,19,21,30,31].

In the past few decades, the impact of tropospheric ozone exposure on plants has been one of the most important environmental issues considered by the European Co-operation Panel on Air Pollution Emissions Control[3]. This has led to a request from policymakers to scientists for methods to quantify these effects.

Earlier experiments with ozone and wheat carried out under semi-natural conditions in open-top chambers (OTCs) and involving several wheat varieties in different countries, such as the U.S.[28], Switzerland[11,14], Finland[24,33], Denmark[1,32], and Sweden[17,24,36], have reported very consistent responses to ozone by crops. These experiments have become the basis for the determination of the critical ozone level by means of the accumulated ozone exposure above a threshold of 40 nl $l^{-1}$·h (nl $l^{-1}$·h = parts per billion h, or ppb h), named ΣAOT40, when the photosynthetic photon flux density (PPFD) is higher than 50 W $m^{-2}$ over a period of 3 months (from anthesis to harvest), which would produce a 5% relative yield loss[16,24,43]. From the regression equation that relates wheat yield to ozone exposure, the ΣAOT40 value predicted was 3,000 nl $l^{-1}$·h and this has been considered as the critical ozone level for crop protection[15].

The use of OTCs may give rise to certain problems, such as a higher ozone absorption by plants due to lower water vapor pressure deficit (VPD), and no watering limitations inside the chamber; soil water limitations lead to lower ozone absorption because of stomatal conductance decline[12,27].

In fact, a problem of overestimation arises when this model is applied for the determination of critical ozone levels when soil water availability is a limiting factor[13,15,20]. It has been explicitly stated that this function cannot be used directly for the estimation of relative economic losses[24,44,45].

There is enough scientific evidence to suggest that the effects of ozone are much more strongly linked to ozone absorption by plants than merely to exposure to ozone[2,22,25,29,35,40]. This is based on the consideration that the uptake of phytotoxic ozone by leaves is strongly controlled by stomatal conductance as well as a significant uptake of ozone by the cuticle, although it does not lead to significant damage[26]. Thus, current critical ozone levels are based on cumulated ozone concentrations, VPD-modified cumulated ozone concentrations, and cumulative ozone uptakes, for a given period, depending on the type of vegetation[45]; the last being only for some agricultural crops (i.e., wheat and potato) and provisionally for sensitive forest trees (i.e., beech and birch). The method employed for calculating accumulated ozone uptake accepted by the CLRTAP was developed by Emberson et al.[9], based on previous work by Jarvis[23], and its application has been checked by several authors on wheat[4,40,41], potato[40,41], clover[35], and forest trees[25,46], among other studies.

Recently, Pleijel et al.[40,41] and Danielsson et al.[4] used the relationships observed between stomatal conductances, obtained in field experiments and inside OTCs, and several meteorological parameters. The aim of their studies was to parameterize a multiplicative model, similar to that developed by Jarvis[23], to model the stomatal conductance for potato (*Solanum tuberosum* L.) and wheat (*Triticum aestivum* L.), and, therefore, to calculate the ozone dose absorbed by these species in different situations of ozone concentration and different environmental conditions inside the OTCs. The values of the ozone-absorbed dose were related to the yield losses observed in those experiments, leading to an estimation of the relative yield losses associated with the dose of accumulated ozone absorbed.

The above authors also evaluated the efficiency of some thresholds of ozone absorption in order to explain the observed yield losses.

The accumulated ozone uptake is calculated by the sum of the hourly stomatal ozone fluxes in a given period, which, in addition to the ozone concentration, depends on the particular species and even on the variety considered. It also depends on environmental factors: the sensitivity of the species to changes (i.e., temperature, relative humidity, VPD, etc.), the phenological stage of the plant, the plant's health condition, etc. In other words, many different factors control stomatal uptake. Nevertheless, in a healthy plant of any given species and/or variety, the main factors on which stomatal conductance and ozone stomatal fluxes (OSF) are dependent are the environmental parameters, such as temperature, radiation, VPD, or soil water potential.

The chamber effect on stomatal uptake can be sorted by using cumulative ozone uptake indices; i.e., determining plant ozone absorption and set dose-response functions instead of exposure-response functions. Dose-response functions can be considered a better indicator than those of exposure-response functions in the assessment of the risk of plant damage[10,13], since the former are more realistic as they consider how environmental factors may affect plant ozone uptake; in particular, how VPD and soil water content (SWC) play a crucial role in the sense that they affect plant stomatal conductance and, hence, the gaseous exchange between the atmosphere and the vegetation, especially in southern Europe where dryer conditions tend to prevail[18].

Various experiments in which wheat plants were exposed to ozone inside OTCs, where a great interannual variability of meteorological conditions was registered, showed a more attenuated response to ozone when employing the accumulated flux indices instead of the exposure-response index, ΣAOT40[39].

## MATERIALS AND METHODS

The development of this work was based on OSF calculations for durum wheat (*T. durum* Desf.), as shown in De la Torre[5], by means of the Y-PLANT model[34]. These fluxes were modeled from stomatal conductance data and environmental data measured at "El Encín" (40º 31' 00'' N, Alcalá de Henares, Madrid), an agroscientific station belonging to IMIDRA (Institute for Rural and Agronomic Research and Development in Madrid), during the years 2001 and 2002.

### Accumulated Ozone Exposure Calculation

The ΣAOT40 index was employed for calculating the accumulated ozone exposure, i.e., the critical level of ozone for the protection of vegetation used during the 1990s in the CLRTAP framework, and currently in use, although the trend is changing towards cumulative absorption indices. Its meaning is the accumulated exposure of $O_3$ over 40 nl $l^{-1}$ during a given time period when the PPFD > 50 W $m^{-2}$[11,24,44].

In 2001, the period from the phenological stage stem elongation until milk development was considered (42 days), and a second one, from anthesis until milk development, with 25 days in total[47]. In 2002, only the period from anthesis until milk development was considered (34 days). In 2001, the period previous to anthesis was considered in order to corroborate the results found by Pleijel et al.[38], showing that the postanthesis periods seem to dominate the grain filling. After the grain filling, the exposure to ozone seems to have no influence in the productivity and, hence, the preanthesis and postgrain filling periods would be irrelevant concerning the effects of ozone exposure on the wheat yield. Moreover, sensitivity to ozone is greater in the periods after anthesis[1,37]. These 25- to 34-day periods contrast with the usual 3-month period employed by these authors; the short periods employed in this study are due to the phenological cycle reduction that the cereals experience in the Mediterranean conditions, as opposed to cereals cultivated in the more humid central and northern Europe.

### Accumulated Flux of Ozone Calculation (AFst_i)

Several indices have been employed for accumulated OSF above a flux rate threshold of "i" nmol of ozone per square meter projected sunlit leaf area, based on hourly values of ozone flux (AFst_i: accumulated flux). These indices have been selected based on previous works of several authors who have studied the interactions of ozone effects and soil water availability on wheat crops[4,9,40,41]. The proposed indices are AFst_4, AFst_5, and AFst_6, i.e., accumulated ozone uptake by leaf area unit, over a certain threshold of 4, 5, or 6 nmol $m^{-2}$ $sec^{-1}$, respectively.

The OSF were calculated by employing the stomatal conductance models shown in De la Torre[5] and following:

$$\text{OSF (nmol m}^{-2}\text{ sec}^{-1}) = D_r\, g_s\, [O_3] \quad (1)$$

where $g_s$, stomatal conductance: mol $m^{-2}$ $sec^{-1}$; $[O_3]$: nmol $mol^{-1}$; $D_r$ = 0.613 (diffusivity rate, $DO_3/DH_2O$).

The accumulated ozone fluxes (AFst_i) were calculated as a sum of the hourly means of the estimated fluxes that were greater than a certain threshold (4, 5, or 6 nmol $m^{-2}$ $sec^{-1}$, respectively, for every AFst_i index previously mentioned) for the time periods described in the former section, as follows:

$$AFst\_i = \Sigma\ (OSF - i\ nmol\ m^{-2}\ sec^{-1}) \quad (2)$$

where "i" is 4, 5, or 6 nmol $m^{-2}$ $sec^{-1}$.

## Calculation of Relative Yield (Y) Losses

Estimations of relative yield losses were accomplished for the two kinds of indices: the accumulated ozone exposure index and the accumulated ozone uptake indices. For this purpose, the empirical relationships proposed by several authors were employed.

For the estimation of the relative yield losses as a function of accumulated ozone exposure, the ΣAOT40 index was employed, following the empirical relationship proposed by Fuhrer et al.[15]:

$$Y = 0.995 - 0.017\ AOT40\ (ppm\ h);\ R^2 = 0.88;\ p < 0.0000[15] \quad (3)$$

Thus, the relative yield loss (%) would be estimated as:

$$Loss\ Y\ (\%) = 100 - (Y * 100) \quad (4)$$

For the estimation of the relative yield loss as a function of the accumulated ozone uptake, several empirical relationships proposed by several authors were employed, in which relative yield (Y) is related to accumulated ozone fluxes (AFst_i):

$$Y = 1 - 0.11\ AFst\_4\ (mmol\ m^{-2});\ R2 = 0.76;\ p < 0.001[9] \quad (5)$$

$$Y = 1 - 0.183\ AFst\_5\ (mmol\ m^{-2});\ R^2 = 0.90;\ p < 0.001[4] \quad (6)$$

$$Y = 0.99 - 0.147\ AFst\_5\ (mmol\ m^{-2});\ R^2 = 0.77;\ p < 0.00001[40] \quad (7)$$

$$Y = 1 - 0.048\ AFst\_6\ (mmol\ m^{-2});\ R^2 = 0.83;\ p < 0.001[41] \quad (8)$$

Thus, the relative yield loss (%) would be estimated following Eq. 4.

## Yield Data

Yield was assessed for two watering regimes: well watered (WW) and nonwatered (NW). Yield was calculated as the production of an area of 4 $m^2$ (g), neglecting the peripheral 1 $m^2$ to avoid the border effect. Yield was calculated as well as the weight per 1000 grains (g).

Differences between WW and NW crops were contrasted by means of an analysis of the variance (ANOVA) for mean yields (STATISTICA, Statsoft, Inc. 1996).

Differences between actual yields for both treatments were contrasted with the differences between estimated yields for both treatments, following the model that best seemed to fit the environmental conditions.

## RESULTS

### Relative Yield Losses in Durum Wheat (*T. durum* Desf. cv. Camacho) in 2001

In 2001, the study of the Camacho wheat commercial variety yield losses were made for the period covered between stem elongation up to milk development, and from anthesis until milk development, as stated previously.

Table 1 shows the values of the employed indices for estimating the relative yield losses for the described periods.

**TABLE 1**
**Values for Each Model-Index Employed for the Calculation of Relative Yield Losses for Durum Wheat (*T. durum* Desf.) cv. "Camacho" in 2001**

| Model-Index | Period | |
|---|---|---|
| | Stem Elongation to Milk Development | Anthesis to Milk Development |
| ΣAOT40 (ppm h)[15] | 2.48 | 1.83 |
| AFst_4 (mmol $m^{-2}$)[9] | 1.65 | 1.39 |
| AFst_5 (mmol $m^{-2}$)[4,40] | 1.25 | 1.11 |
| AFst_6 (mmol $m^{-2}$)[41] | 0.92 | 0.86 |

The index values (see Table 1) were employed for estimating the yields ($Y_i$) and relative yield losses (Loss $Y_i$), following the respective relationships "Y vs. $INDEX_i$" (see Eqs. 3–8). The obtained values of estimated relative yield losses are shown in Table 2 and Fig. 1.

**TABLE 2**
**Estimation of Relative Yield Losses (%) for Two Different Phenological Periods and Several Applied Models for Durum Wheat (*T. durum* Desf.) cv. "Camacho" in 2001**

| Model Applied | Period | |
|---|---|---|
| | Stem Elongation to Milk Development | Anthesis to Milk Development |
| ΣAOT40 (ppm h)[15] | 5 | 4 |
| AFst_4 (mmol $m^{-2}$)[9] | 18 | 15 |
| AFst_5 (mmol $m^{-2}$)[4] | 23 | 20 |
| AFst_5 (mmol $m^{-2}$)[40] | 19 | 17 |
| AFst_6 (mmol $m^{-2}$)[41] | 4 | 4 |

### Relative Yield Losses in Durum Wheat (*T. durum* Desf. cv. Camacho) in 2002

A new variable in the water treatment was introduced in 2002 from milk development phenology: WW wheat (up to field capacity) and NW wheat. Table 3 shows the calculated values corresponding to the four indices employed for estimating the relative yield losses (see "Accumulated Ozone Exposure Calculation" and "Accumulated Flux of Ozone Calculation" above).

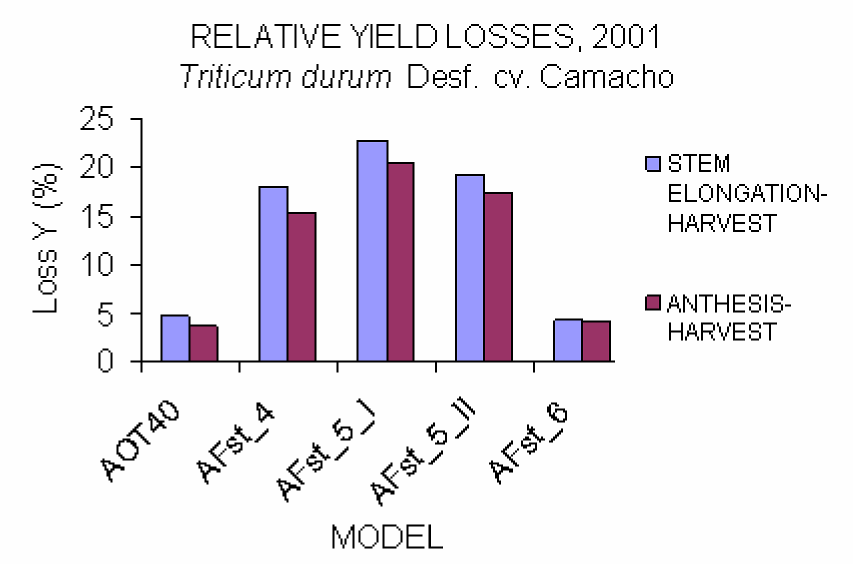

**FIGURE 1.** Relative yield losses (%) estimation for *T. durum* cv. Camacho exposed to ozone in 2001, in two periods, stem elongation to harvest and anthesis to harvest. Estimations based on ΣAOT40[15] and AFst_i[4,9,40,41].

**TABLE 3**
**Values for Each Model-Index Employed for the Calculation of Relative Yield Losses for Durum Wheat (*T. durum* Desf.) cv. "Camacho" in 2002 for a Time Exposure of 816 h**

| Treatment | Model Applied | | | |
|---|---|---|---|---|
| | ΣAOT40 (ppm h)[15] | AFst_4 (mmol $m^{-2}$)[9] | AFst_5 (mmol $m^{-2}$)[4,40] | AFst_6 (mmol $m^{-2}$)[41] |
| NW | 7.32 | 1.58 | 1.25 | 0.94 |
| WW | 7.32 | 2.42 | 2.03 | 1.67 |

Relative yield losses induced by environmental ozone exposure were calculated by applying these values to Eqs. 3, 5, 6, 7, and 8, respectively. Each of them was calculated for the different indices from Table 3. Table 4 and Fig. 2 show the relative yield losses associated with each index and water treatment.

**TABLE 4**
**Estimation of Relative Yield Losses (%) for Two Different Water Treatments and Several Applied Models for Durum Wheat (*T. durum* Desf.) cv. "Camacho" in 2002**

| Water Treatment | Model Applied | | | | |
|---|---|---|---|---|---|
| | ΣAOT40 (ppm h)[15] | AFst_4 (mmol $m^{-2}$)[9] | AFst_5 (mmol $m^{-2}$)[4] | AFst_5 (mmol $m^{-2}$)[40] | AFst_6 (mmol $m^{-2}$)[41] |
| NW | 15 | 17 | 23 | 19 | 5 |
| WW | 15 | 27 | 37 | 31 | 8 |

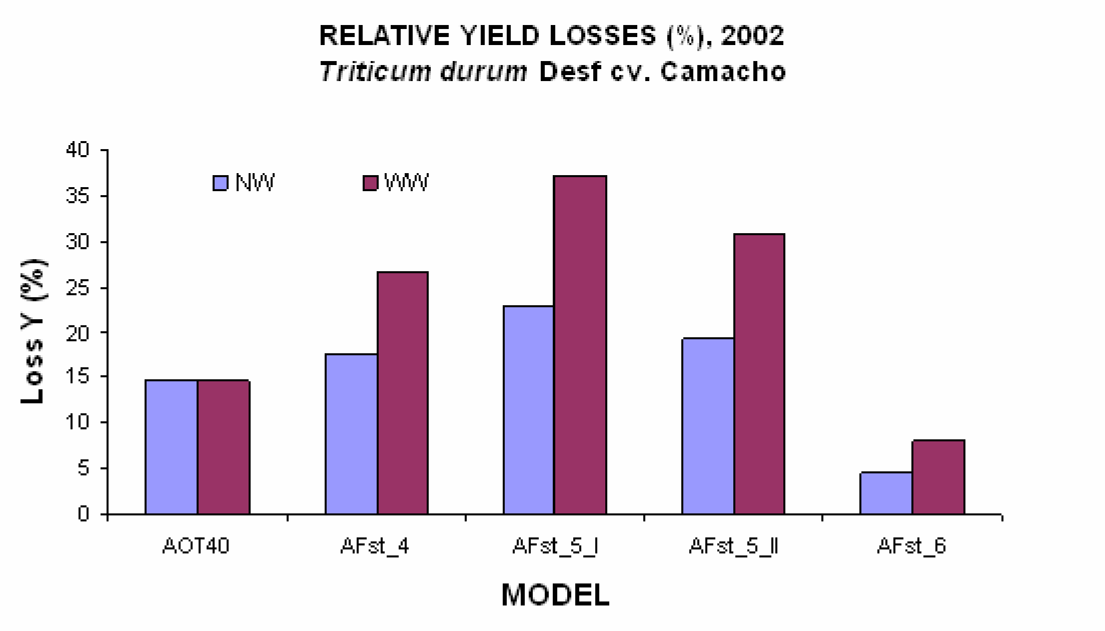

**FIGURE 2**. Relative yield losses (%) estimation for *T. durum* cv. Camacho exposed to ozone in 2002, for two water treatments. Estimations based on ΣAOT40[15] and AFst_i[4,9,40,41].

## Relative Yield Loss Differences between Water Treatments

Table 5 shows the difference between water treatments (NW vs. WW) for the estimation of relative yield losses and for every model applied.

**TABLE 5**
**Differences in the Estimated Relative Yield Losses (%) between Water Treatments for the Anthesis to Milk Development Phenological Period in 2002, for every AFst_i-Based Model**

| Model Applied | | | |
|---|---|---|---|
| **AFst_4[9]** | **AFst_5[4]** | **AFst_5[40]** | **AFst_6[41]** |
| 10% | 14% | 2% | 3% |

In Table 4 and Fig. 2, it was already shown that the estimated relative yield losses were always higher for WW plants than for NW plants, regardless of the accumulated ozone uptake–based model applied[4,9,40,41].

## DISCUSSION

### Relative Yield Losses in Durum Wheat (*T. durum* Desf. cv. Camacho) in 2001

The results (see Table 2 and Fig. 1) prove that there are almost no differences in the relative yield losses (“Loss Y”) when comparing the two considered periods (stem elongation to milk development vs.

anthesis to milk development). There is only a difference of 1%, considering the accumulated exposure of ozone over the 40 ppm h–based model (ΣAOT40), and differences of 0–3% when applying the accumulated stomatal flux models (AFst_i), depending on the employed model.

These minimum differences in the relative yield losses, corresponding to a difference of 17 days in the exposing time period, are due to the high environmental ozone concentrations registered from anthesis as a consequence of the elevated temperatures and sun radiation. That is to say, the ΣAOT40 or the AFst_i accumulated during these 17 days are negligible compared to the ΣAOT40 or the AFst_i accumulated in the anthesis to milk development phenological periods. In fact, more than 80% of the total previous exposure is due to the plant exposure to ozone from anthesis, independently of the considered index for its calculation, excepting for AFst_6. This index indicates that most of the OSF took place at the end of the cultivar life cycle, as 100% of theses fluxes above the threshold of 6 nmol $m^{-2}$ were registered between anthesis and milk development. Thus, it could be much more phytotoxic than predicted because it seems to be an OSF threshold from which the plant detoxification capacity is exceeded[6]. Moreover, several authors[1,37,38] have remarked that it is, indeed, in the anthesis to milk development period when cereals are especially sensitive to ozone, as the photoassimilates begin to transport mainly up to the spike in order to enable grain development, producing an increment in the plant stomatal conductance and physiological activity.

Relative yield losses calculated by means of the ΣAOT40 index[15] were about 4–5%. These same relative yield losses, calculated by means of the accumulated ozone uptake indices AFst_4 and AFst_5, were between 11 and 18 points higher than those calculated with ΣAOT40, depending on the AFst_i index and the period considered. Nevertheless, the AFst_6 index showed an estimation of relative yield losses of 4% (see Table 2 and Fig. 1). The estimation provided by AFst_4 and AFst_5 indices would be an excessive overestimation of real losses, as the ΣAOT40 index sometimes provides an overestimation (depending on the temperature, VPD, and soil moisture values)[15]. Having this fact in mind, the AFst_6 index would provide the most accurate estimation, as it involves physiological parameters (as stomatal conductance) as well as the environmental factors that modulate the ozone absorption[39]. There would also be a biological mechanism involved in the reduction of the estimated relative yield losses by means of the AFst_6 index in regards to the ΣAOT40 index; it seems to be an ozone uptake threshold that needs to be exceeded in order to register plant damages that would induce yield losses. This threshold would be around 6 nmol $m^{-2}$ $sec^{-1}$[6]. Once that threshold is exceeded, an ozone detoxification mechanism by means of ascorbic acid is started[6].

The results indicate that relative yield losses estimated from accumulated ozone fluxes (AFst_6) for Mediterranean wheat cultivars (*T. durum* Desf. cv. Camacho) could be around 4%, indepent of the time period considered, as AFst_6 index values indicate that the accumulated ozone uptake before anthesis would not have any significative influence in the relative yield losses (see Table 2 and Fig. 1). Thus, the real absorbed dose of ozone is what really deserves importance; the environmental concentration of ozone or the exposure time to ozone are not so important if they do not imply elevated ozone absorption during the period of high sensitivity.

## Relative Yield Losses in Durum Wheat (*T. durum* Desf. cv. Camacho) in 2002

In 2002, the ΣAOT40 index shows an estimation of relative yield losses of 15%, i.e., 9% higher than the one for 2001; this is due to a quite more elevated accumulated ozone exposure in 2002 (see Tables 1 and 3). Nevertheless, when comparing the relative yield losses estimated by means of the AFst_i indices for both years at the anthesis to milk development phenological period, it can be seen that they are very similar, just between 1–3% higher for 2002 (depending on the applied model). Relative yield losses estimated by means of the AFst_6 index are almost identical as well, with 4% in 2001 and between 5 and 8% in 2002, depending on the water treatment applied (see Tables 1 and 3). This fact indicates that indices based on accumulated ozone uptake, besides being more realistic because they integrate environmental and physiological variables[39], seem to better explain the meteorological variability registered in the different sampled years and, thus, they explain more realistically the meteorological influence in the ozone phytotoxicity.

These results are according to De la Torre and Sierra[7], who found relative yield losses for *T. aestivum* of 9–11%, applying the ΣAOT40 index[15], grown in similar Mediterranean conditions in Catalonia (Spain) (i.e., *Secans Semifrescals* commercial variety). In that study, De la Torre and Sierra[7] also found minimum relative yield losses when soil water availability was lower.

## Comparison of the Models Employed for the Estimation of Relative Yield Losses

The ΣAOT40 index is based on environmental concentrations of ozone accumulated during a certain period (anthesis to milk development) and, thus, it would be the maximum accumulated ozone uptake by the plant. This index would then determine the maximum relative yield losses. Since AFst_i indices involve biotic factors (stomatal conductance, plant physiological activity, etc.) as well as environmental factors in the ozone uptake modulation, the estimation of relative yield losses employing these indices should be equal to or lower (but never higher) than the estimations employing the ΣAOT40 index; this is because the AFst_i indices consider that the ozone uptake can be lower due to partial stomatal closure, phenological stage, or bad plant health condition.

The interpolation of the accumulated ΣAOT40 value (see Table 3) in the regression line “Y vs. ΣAOT40” proposed by Fuhrer et al.[15] indicates that this value would be in an intermediate range, corresponding to values registered in OTCs with nonfiltered air in Switzerland or the U.S.[15]. With this in mind, the application of this model would be adequate for Mediterranean conditions, even though the inconvenience of its application to the relative yield loss calculation has been mentioned before (see Introduction[44]).

Several trends were found when interpolating the values for AFst_i indices (see Table 3) in the corresponding regression lines[4,9,40,41].

AFst_5 values interpolated from the Danielsson et al.[4] regression line were similar to those experimental values from ozone-fumigated chambers (ozone concentrations above the environmental concentrations) employed to build the model[4] (shown at the right end of the graph in Danielsson et al.[4]). Here, minimum yields and relative yield losses of about 23–37% are shown (see Table 4 and Fig. 2), which are too high, as relative yield losses corresponding to the ΣAOT40 index are about 15%[15].

When interpolating the values obtained for AFst_4 and AFst_5 from the respective regression lines from Emberson et al.[9] and Pleijel et al.[40], they were similar to those experimental values employed to build the models obtained with plants in OTCs with nonfiltered air or at environmental conditions (outside chambers) (shown at the middle-right of the graphs[9,40]). Here, the estimated relative yield losses were still too high (17–31%) regarding the ΣAOT40 model (15%)[15](see Table 4 and Fig. 2).

Nevertheless, the AFst_6 model[41] provided minimum relative yield losses, between 5 and 8% (see Table 4 and Fig. 2), similar to those experimental values employed to build the regression line with plants in OTCs with nonfiltered air or at environmental ozone concentrations[41], corresponding to elevated yields (shown at the top left of the regression line, Pleijel et al.[41]).

Thus, the AFst_6 index[41] (see Table 4 and Fig. 2) would be the model based on accumulated ozone uptake that would most accurately estimate relative yield losses for wheat (5–8%), which were below those estimated with the ΣAOT40 index, based on accumulated ozone exposures (15%). The AFst_6 index[41] proved to be the most accurate index for 2001 as well (see Discussion).

As Table 6 shows, the obtained AFst_6 values were mainly due to high ozone registered concentrations: the environmental ozone concentrations were above 40 ppb for 72% of AFst_6 data (NW treatment) and they were above 40 ppb for 99% of AFst_6 data (WW treatment), indicating that the main factor to get high OSF is the elevated environmental ozone concentrations. Nevertheless, it depends greatly on the water treatment, as equal or even greater AFst_6 values were obtained in this experiment during a 30-day period compared to the experiments performed in central and northern Europe during a 3-month period[41].

**TABLE 6**
**AFst_6 Values (%) with Ozone Concentrations Higher than 40 ppb**

| Treatment | |
|---|---|
| **NW** | **WW** |
| 72% | 99% |

## Relative Yield Loss Differences between Water Treatments

Differences in relative yield losses due to an increase in the soil water availability vary depending on the chosen model (see Table 6). Nevertheless, since AFst_6 seems to be the most accurate model (see Discussion)[41], these differences would be about 3%, i.e., wheat crops watered up to field capacity would have 3% more relative yield losses than NW wheat crops. Although 3% would seem a small difference, in 2002 there was rain until the end of anthesis; the water treatment was only applied for 34 days at the end of the phenological period, when stomatal conductances are lower, implying that if soil water availability differences had been higher during a longer period, these differences in the relative yield losses could have been much higher because of the water treatment. This result is in accordance with results from Fuhrer[12] and Khan and Soja[27], who found that an increment in soil water availability makes wheat plants more sensitive to ozone effects, inducing yield losses due to an increase in the stomatal conductance.

## Comparing the Results with Real Yield Data

The relative yield losses obtained from the application of the AFst_6 model[41], i.e., the one that applied most accurately to the environmental conditions of this study, were compared to the real wheat yield data (see in Table 7), expressed as mean yield (g/4 $m^2$) and weight per 1,000 grains (g). The watering treatment favors a higher yield compared to the NW treatment, although differences were not significative, indicating that soil water availability was minimum. The results found in this study show that lower yield (higher relative yield losses) was found in the WW crop; differences due to the water regime would probably be greater if pluviometry at the end of anthesis would have been lower, so the increment in relative yield losses due to ozone uptake for the WW crop was surely compensated by a better soil water availability.

## CONCLUSIONS

The results obtained show that relative yield losses in wheat due to ozone exposure seem to be much more strongly linked to the real quantity of ozone absorbed by plants (calculated by means of the AFst_i indices) than to the environmental concentration of ozone (ΣAOT40 index).

From the analysis of several AFst_i indices, AFst_6 seems to be the most accurate for the estimation of relative yield losses for wheat crops: an accumulated ozone uptake–based index above a threshold of 6 nmol $m^{-2}$ $sec^{-1}$ during the anthesis to harvest phenological period.

Relative yield loss calculations were higher in NW crops in just a 1-month period (34 days), with 3% relative yield losses, more than in crops watered until field capacity during that same period; this is according to several authors who found that an increment in soil water availability at the anthesis to harvest period make plants more sensitive to ozone effects due to an increment in the plant transpiration, and thus in the stomatal conductance, provoking a higher yield loss.

**TABLE 7**
**Real Yield, Expressed as Mean Yield (g) and Weight per 1,000 grain (g) for both Water Treatments**

| Water Treatment | | | | *p* Value |
|---|---|---|---|---|
| WW | | NW | | |
| Mean Yield (g/4 $m^2$) | 1,000 Grain Weight (g) | Mean Yield (g/4 $m^2$) | 1,000 Grain Weight (g) | |
| 3378 ± 329 | 56 ± 1.63 | 2944 ± 349 | 53 ± 0.65 | n.s |

*Note:* *p* value is obtained from the applied ANOVA to calculate the differences in wheat yield between water treatments, with a confidence level of 95% ($p < 0.05$).

In Mediterranean conditions, with elevated sun radiation and elevated temperatures, which are indeed factors needed for ozone photochemical formation, the ozone concentrations registered during the anthesis to harvest period are quite high and they would be expected to produce quite more elevated relative wheat yield losses than they do, as estimated by means of the AFst_6 index; in fact, they decrease from about 15% (as calculated by means of the ΣAOT40 index) to 5–8%, depending on the watering regime (as calculated by means of the AFst_6 index).

## ACKNOWLEDGMENTS

Acknowledgement is given to TheScientificWorldJOURNAL Editor responsible for handling this manuscript. Special thanks are given to Dra. Agueda González Rodriguez (IMIDRA) for her intermediation with her research institute in order to be able to make the experimental measurements at El Encin. Last, but not least, thanks are given to Elisa and Santiago de la Torre and Jordan De Mello for their great help with language revision.
